# Mechanisms regulating PD-L1 expression on tumor and immune cells

**DOI:** 10.1186/s40425-019-0770-2

**Published:** 2019-11-15

**Authors:** Shuming Chen, George A. Crabill, Theresa S. Pritchard, Tracee L. McMiller, Ping Wei, Drew M. Pardoll, Fan Pan, Suzanne L. Topalian

**Affiliations:** 1 Department of Surgery, Johns Hopkins University School of Medicine, Sidney Kimmel Comprehensive Cancer Center, and Bloomberg~Kimmel Institute for Cancer Immunotherapy, Baltimore, MD 21287 USA; 2 Department of Oncology, Johns Hopkins University School of Medicine, Sidney Kimmel Comprehensive Cancer Center, and Bloomberg~Kimmel Institute for Cancer Immunotherapy, Baltimore, MD 21287 USA

**Keywords:** PD-L1, Cytokines, Interferon gamma, Interleukins, Tumor microenvironment, Transcription factors, Cancer immunotherapy

## Abstract

**Background:**

The PD-1/PD-L1 checkpoint is a central mediator of immunosuppression in the tumor immune microenvironment (TME) and is primarily associated with IFN-g signaling. To characterize other factors regulating PD-L1 expression on tumor and/or immune cells, we investigated TME-resident cytokines and the role of transcription factors in constitutive and cytokine-induced PD-L1 expression.

**Methods:**

Thirty-four cultured human tumor lines [18 melanomas (MEL), 12 renal cell carcinomas (RCC), 3 squamous cell carcinomas of the head and neck (SCCHN), and 1 non-small-cell lung carcinoma (NSCLC)] and peripheral blood monocytes (Monos) were treated with cytokines that we detected in the PD-L1+ TME by gene expression profiling, including IFN-g, IL-1a, IL-10, IL-27 and IL-32g. PD-L1 cell surface protein expression was detected by flow cytometry, and mRNA by quantitative real-time PCR. Total and phosphorylated STAT1, STAT3, and p65 proteins were detected by Western blotting, and the genes encoding these proteins were knocked down with siRNAs. Additionally, the proximal promoter region of *PDL1* (*CD274*) was sequenced in 33 cultured tumors.

**Results:**

PD-L1 was constitutively expressed on 1/17 cultured MELs, 8/11 RCCs, 3/3 SCCHNs, and on Monos. Brief IFN-g exposure rapidly induced PD-L1 on all tumor cell lines and Monos regardless of constitutive PD-L1 expression. PD-L1 mRNA levels were associated with protein expression, which was diminished by exposure to transcriptional inhibitors. siRNA knockdown of STAT1 but not STAT3 reduced IFN-g- and IL-27-induced PD-L1 protein expression on tumor cells. In contrast, STAT3 knockdown in Monos reduced IL-10-induced PD-L1 protein expression, and p65 knockdown in tumor cells reduced IL-1a-induced PD-L1 expression. Notably, constitutive PD-L1 expression was not affected by knocking down STAT1, STAT3, or p65. Differential effects of IFN-g, IL-1a, and IL-27 on individual tumor cell lines were not due to *PDL1* promoter polymorphisms.

**Conclusions:**

Multiple cytokines found in an immune-reactive TME may induce PD-L1 expression on tumor and/or immune cells through distinct signaling mechanisms. Factors driving constitutive PD-L1 expression were not identified in this study. Understanding complex mechanisms underlying PD-L1 display in the TME may allow treatment approaches mitigating expression of this immunosuppressive ligand, to enhance the impact of PD-1 blockade.

## Background

Programmed death ligand 1 (PD-L1, CD274) expressed on tumor and/or immune cells in the tumor microenvironment (TME) interacts with PD-1 on tumor infiltrating lymphocytes, attenuating effector T cell responses and allowing tumors to escape immune attack [1, 2]. Understanding how TME-resident cytokines and signaling pathways regulate PD-L1 expression may provide therapeutic opportunities to mitigate PD-L1-induced intratumoral immunosuppression [3].

There are two general mechanisms by which tumor cells can express PD-L1, protecting them from immune elimination: “innate immune resistance” and “adaptive immune resistance” [4]. Innate resistance refers to constitutive PD-L1 expression on tumor cells, resulting from *PDL1* gene amplification or aberrant activation of oncogenic signaling pathways. Activation of ALK/STAT3 in T cell lymphoma [5], AP-1/JAK/STAT in classical Hodgkin lymphoma (cHL) [6], the microRNA-200/ZEB1 axis in non-small-cell lung cancer (NSCLC) [7], c-jun/STAT3 in BRAF inhibitor-resistant melanoma [8], and PI3K in glioma [9] have each been reported to upregulate PD-L1 expression on tumor cells. Additionally, Myc has been shown to regulate constitutive PD-L1 expression at the mRNA level in multiple tumors, such as T cell acute lymphoblastic leukemia, melanoma and NSCLC [10]. Recently, post-transcriptional regulation of PD-L1 has also attracted attention, with reports that cyclin-dependent kinase-4 (CDK4) and glycogen synthase kinase 3 beta (GSK3B) can promote PD-L1 protein degradation in cultured tumors [11, 12].

In contrast to innate resistance, adaptive immune resistance refers to PD-L1 expression on tumor or immune cells in response to inflammatory factors secreted in the TME during antitumor immune responses. While IFN-g is generally thought to be the primary T cell derived cytokine responsible for adaptive PD-L1 expression, we have described several additional TME-resident cytokines that can upregulate PD-L1 expression on cultured human monocytes (Monos) and/or tumor cells, including IL-1a, IL-10, IL-27 and IL-32 g [13–15]. Transcripts for IFN-g, IL-10 and IL-32 g were over-expressed in PD-L1+ compared to PD-L1(−) melanoma biopsies; in vitro, IL-10 and IL-32 g induced PD-L1 expression on Monos but not on melanoma cells [15]. IL-1a was upregulated in Epstein-Barr virus (EBV) negative PD-L1+ cHL, and IL-27 was upregulated in EBV+ PD-L1+ cHL. When combined with IFN-g, IL-1a and IL-10 further increased PD-L1 protein expression on human Monos in vitro, compared to the effects of IFN-g alone. IL-27 increased PD-L1 expression on Monos as well as dendritic cells, T cells, and some tumor cell lines [14, 16] . Others have reported that the transcription factors JAK/STAT1 [17], IRF-1 [18] and NF-kB [19], involved in inflammatory cytokine production, can contribute to IFN-g-induced PD-L1 expression on hematopoietic tumors, lung cancer, and melanoma, respectively. In a murine medulloblastoma model, the cyclin-dependent kinase CDK5 appeared to regulate IFN-g-induced PD-L1 expression [20]. Overall, existing evidence suggests that PD-L1 may be differentially regulated with respect to specific signaling pathways and transcription factors in different cell types, although IFN-g appears to be a dominant cytokine driving expression of this immunosuppressive ligand.

We undertook the current study to broadly examine mechanisms underlying constitutive and cytokine-induced PD-L1 expression in four human tumor types – melanoma (MEL), renal cell carcinoma (RCC), squamous cell carcinoma of the head and neck (SCCHN), and NSCLC – and to investigate the potential roles of STAT1, STAT3, and p65 activation in driving constitutive and inducible PD-L1 expression on tumor cells and Monos.

## Methods

### Cell culture and flow cytometry

Established cultures of human MELs, RCCs, SCCHNs, and NSCLC (Additional file 5: Table S1) were maintained in RPMI 1640 medium or DMEM with 10% heat-inactivated fetal calf serum. Human Monos were enriched by negative selection from cryopreserved peripheral blood mononuclear cells with the Pan Monocyte Isolation Kit (Miltenyi Biotec, San Diego, CA). Cells were cultured in the presence of recombinant IFN-g (100 or 250 IU/ml; Biogen, Cambridge, MA), IL-1a (10 ng/ml), IL-6 (20 ng/ml), IL-10 (100 ng/ml), IL-27 (50 ng/ml) or IL-32 g (100 ng/ml; all R&D Systems, Minneapolis, MN) for the indicated time periods (Additional file 6: Table S2). In some experiments, actinomycin D (ActD, 10 μg/ml) or cycloheximide (CHX, 2 μg/ml; both Thermo Fisher Scientific, Waltham, MA) was added to cultures 1 h before IFN-g treatment. Adherent cells were harvested with trypsin. To assess cytokine effects on PD-L1 expression, cells were stained with anti-human PD-L1 (clone MIH4, ThermoFisher Scientific, Carlsbad, CA) or an isotype control. HLA-DR (clone L243, Becton Dickenson, San Jose, CA) staining was performed simultaneously to provide a control for the effects of IFN-g. PD-L2 was stained with clone MIH18 (Thermo Fisher Scientific). Data were acquired on the BD FACSCalibur and analyzed with FlowJo Software (TreeStar, Ashland, OR). Expression level of a molecule was calculated as delta mean fluorescence intensity (∆MFI), which is MFI of specific staining – MFI of isotype control staining. Cytokine-induced expression of a molecule was calculated as ∆∆MFI, which is ∆MFI with cytokine exposure – ∆MFI without cytokine exposure.

### Real time quantitative reverse transcriptase PCR (qRT-PCR)

mRNA was extracted from cells 6–16 h after cytokine treatment with the RNeasy Mini Kit (QIAGEN, Germantown, MD). Total mRNA from each sample was reverse-transcribed with the qScript™ cDNA SuperMix (Quanta Bioscience, Beverly, MA). Real-time PCR was performed in triplicate for each sample using commercial primers and probes for *CD274, HLA-DRA*, and housekeeping genes (Thermo Fisher Scientific). Forty cycles of PCR were conducted using a QuantStudio 12 K Flex Real-Time PCR System. Results were analyzed using the manufacturer’s software (Applied Biosystems). Fold change of mRNA expression before and after cytokine treatment was calculated as 2^(ΔCt _before_ – ΔCt _after_), in which ΔCt = Ct _specific probe_ – Ct _internal control_.

### Western blotting

Lysates of whole cells or nuclear proteins were prepared with M-Per and NE-Per (Thermo Fisher Scientific) respectively, as described [15]. Briefly, 20 μg protein per lane was separated by 4–12% Bis-Tris SDS-PAGE under reducing conditions and transferred to a polyvinylidene difluoride membrane, which was blocked with 5% dry non-fat milk. Membranes were stained with antibodies specific for signal transducer and activator of transcription (STAT)1 (polyclonal, catalog # 9172), phospho-STAT1 (clone 58D6), STAT3 (clone 124H6), phospho-STAT3 (pSTAT3; clone M9C6), p65 (clone D14E12), phospho-p65 (pp65; clone 93H1), c-jun (clone 60A8) and phospho-c-jun (pc-jun; clone D47G9) (all Cell Signaling Technology, Beverly, MA) at 4 °C overnight. Membranes were counterstained with anti-rabbit IgG-HRP (1:1000–1:12,000 dilution) or anti-mouse IgG-HRP (1:1000–1:5000) for 1 h at room temperature (GE Healthcare, UK or Kindle Bioscience, Greenwich, CT). Blots were also stained with anti-beta-actin-peroxidase (1:200,000 dilution; Sigma, St. Louis, MO, clone AC-15). Proteins were detected by ECL Western blotting detection reagents (GE Healthcare) or Hi/Lo Digital–ECL Western Blot Detection Kit (Kindle Bioscience) and the density of the target molecule was quantified with the ImageJ program (https://imagej.nih.gov/ij/) [21]. Normalized density was calculated as the ratio of target molecule density to beta-actin density.

### Short inhibitory RNA (siRNA) transfection

ON-TARGET plus SMART pool siRNAs for STAT1, STAT3, and p65 were purchased from Dharmacon (Lafayette, CO). siRNA transfection was done with the Nucleofector II or 4D-nucleofector device (Lonza, Basel, Switzerland) following the Amaxa Cell Line Nucleofector Kit, Human Monocyte Nucleofector Kit, or SF/SE Cell Line 4D Nucleofector X kit protocols. Briefly, 1 × 10^6^–4 × 10^6^ tumor cells or 1 × 10^7^ Monos were suspended in 100 μl transfection solution supplemented with 100–300 pmol specific or scrambled siRNA. Electroporation was done with transfection programs recommended in the Lonza Knowledge Center (https://knowledge.lonza.com/) [22]. Two days after transfection, cells were incubated with cytokines. Knockdown effects and transcription factor phosphorylation were detected 15 min later by Western blotting. Percentage of knockdown was calculated based on the actin-normalized density of the target molecule in Western blotting, by the formula (scrambled siRNA - specific siRNA)/scrambled siRNA × 100. The average targeted knockdown achieved in this study was 70%. PD-L1 and HLA-DR expression at the cell surface was detected and quantified 24 h later by flow cytometry, and the effects of knockdown with target-specific siRNAs were calculated with reference to scrambled siRNA.

### PDL1 promoter region sequencing

Genomic DNA from cultured tumor cell lines or cryopreserved peripheral blood lymphocytes was extracted from 1 × 10^6^ cells using the PureLink Genomic DNA kit (Thermo Fisher Scientific, K1820–00). Based on the public *PDL1 (CD274)* gene sequence (GenBank NC_000009.12), three primers (PDLP-F1, 5’GTTTCCAGGCATCACCAGATGCT; PDLP-F2, 5’TCCTCATGGGTTATGTGTAGTTTG; PDLP-R, 5’CCTCATCTTTCTGGAATGCCCTA) were designed to amplify 2.1 kb and 1.1 kb regions that are immediately upstream of the ATG translation start site. These two regions were amplified using an Expand TM High Fidelity PCR system (Sigma, catalog # 11732650001). Amplified PCR products were purified by a QIAquick PCR Purification kit (Qiagen, catalog # 28104) and sent to the Johns Hopkins University Core Facility for Sanger sequencing. Amplicons were sequenced using the following primers: PDLP-seq, 5’TGCTGAATTCAGTCCTTAATGG and PDLP-seqR, 5’CCATTAAGGACTGAATTCAGCA; PDLP-seq2, 5’CAGATACTCTGGAAGAGTGGCT and PDLP-seq2R, 5’AGCCACTCTTCCAGAGTATCTG.

## Results

### IFN-g-induced PD-L1 protein expression on tumor cells is associated with de novo PD-L1 (CD274) mRNA transcription

**Fig. 1:**
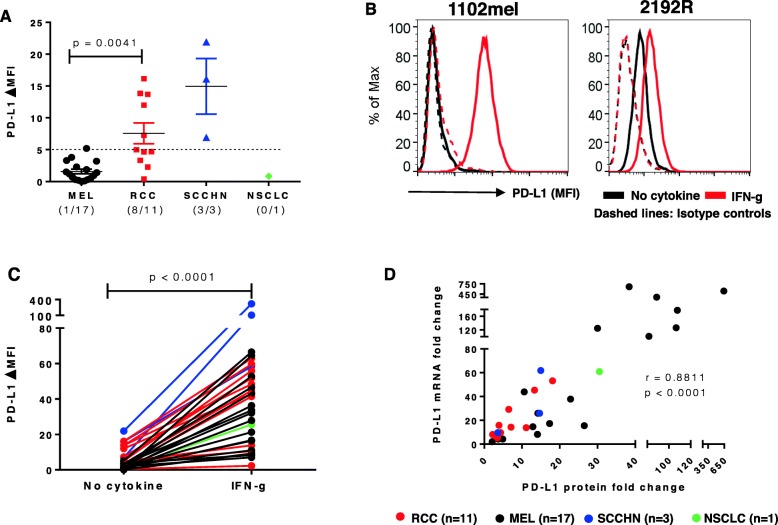
IFN-g-induced PD-L1 protein expression is associated with new *PDL1* mRNA transcription in 32 cultured human tumors. **a.** Constitutive expression of cell surface PD-L1 protein by select tumor lines, detected by flow cytometry. RCCs expressed significantly more PD-L1 than MELs (*p* = 0.0041). Kruskal-Wallis test (Dunn’s multiple comparisons test), 2-sided *p*-value. ΔMFI, mean fluorescence of specific staining – isotype staining. Cell lines with ∆MFI ≥ 5, indicated by horizontal dotted line, were considered to be PD-L1 positive. **b.** Representative examples of IFN-g-induced (left panel) or IFN-g-enhanced (right panel) PD-L1 protein expression. Cultured tumor cells (1102mel, melanoma; 2192R, RCC) were treated with IFN-g 250 U/ml for 48 h, then cell surface PD-L1 protein was detected by flow cytometry. Histograms from two representative cell lines with or without constitutive PD-L1 expression are shown. **c.** IFN-g significantly increased PD-L1 protein expression on all types of tumor cells tested. Wilcoxon matched-pairs signed rank test, 2-sided p-value. **d.** IFN-g-induced PD-L1 protein expression is significantly associated with new *PDL1* mRNA transcription. Thirty-two cultured tumor lines were treated with IFN-g 250 U/ml. PD-L1 mRNA and cell surface protein expression were detected by qRT-PCR and flow cytometry after 14 h and 48 h, respectively. Fold changes in PD-L1 protein (ΔMFI) and mRNA (ΔCt) were calculated, compared to pretreatment values. Spearman correlation r value, 2-sided p-value. A, C and D, data combined from 3 separate experiments

We first assessed constitutive tumor cell surface PD-L1 protein expression with flow cytometry on 32 established tumor lines, including 17 MELs, 11 RCCs, 3 SCCHNs and 1 NSCLC. PD-L1 was not constitutively expressed on 16 of 17 cultured MELs, nor on one NSCLC; in contrast, 8 of 11 RCCs and 3 of 3 SCCHNs constitutively expressed PD-L1 on the cell surface (Fig. 1a). The absence of constitutive expression on melanoma cell lines contrasts with a previous report [23]. Regardless of baseline PD-L1 expression, all four tumor types showed significantly enhanced PD-L1 protein expression after brief exposure to IFN-g (*p* < 0.0001; Fig. 1b and c) [15]. Cell surface expression of CD119 (IFN-g receptor 1), the ligand-binding alpha chain of the heterodimeric IFN-g receptor, was assessed with flow cytometry on 28 of 32 cell lines and was compared to IFN-g-enhanced PD-L1 protein expression. Although CD119 was detected in each cell line, CD119 levels did not correlate with the magnitude of increased PD-L1 expression after IFN-g exposure (Spearman correlation test, r = 0.19, *p* = 0.32; data not shown), suggesting that even low levels of CD119 are sufficient for signal transduction. To investigate whether induction of PD-L1 protein was associated with new synthesis of *PDL1* mRNA, changes in mRNA and protein levels were examined in 32 tumor cell lines representing four cancer types, before and after IFN-g treatment. Changes in *PDL1* mRNA expression correlated significantly with PD-L1 cell surface protein expression (*p* < 0.0001; Fig. 1d). These results suggest that IFN-g activates factors promoting new *PDL1* mRNA transcription. In some cell lines, IFN-g also induced or enhanced tumor cell surface expression of PD-L2, the second ligand for PD-1, although these levels were substantially lower than for PD-L1 (Additional file 7: Table S3).

To further explore this phenomenon, we incubated cultured MELs with ActD, a mRNA transcription inhibitor, or CHX, a protein synthesis inhibitor, prior to IFN-g exposure. Six h after IFN-g exposure, we found that each chemical completely blocked the emergence of PD-L1 protein on the cell surface. As expected, in the same cells, ActD suppressed IFNg-induced *PDL1* mRNA transcription while CHX did not (Additional file 1: Figure S1). These data suggest that IFN-g drives new PD-L1 transcription and translation, and that translocation of preexisting intracellular PD-L1 protein stores is not a major mechanism underlying IFN-g-induced PD-L1 expression on the cell surface.

### STAT1 but not STAT3 mediates IFN-g-induced PD-L1 protein expression on tumor cells

**Fig. 2:**
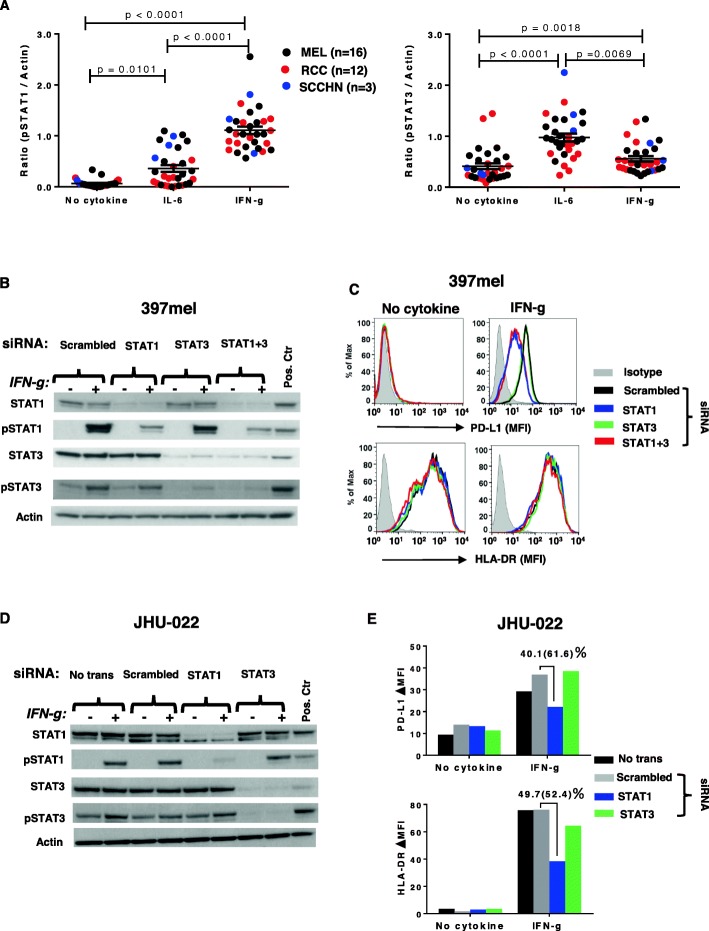
STAT1, but not STAT3 phosphorylation is necessary for IFN-g-induced PD-L1 protein expression on tumor cells. **a.** IFN-g had a major effect on STAT1 phosphorylation (left panel) but only a minor effect on STAT3 phosphorylation (right panel) in 31 tumor cell lines tested, including MELs, RCCs, and SCCHNs. IL-6 had a reciprocal effect in the same cell lines. Cultured cells were treated with IFN-g 250 U/ml or IL-6 20 ng/ml. Cells were harvested after 15 min and phosphorylation of STAT1 and STAT3 was detected by Western blotting. Protein bands were quantified by ImageJ and results were normalized to beta-actin expression. Kruskal-Wallis test (Dunn’s multiple comparisons test), 2-sided *p*-values. **b** and **c.** Specific siRNA knockdown of STAT1, but not STAT3 mRNA expression in 397mel cells significantly reduced total and phosphorylated STAT1 proteins and reduced IFN-g-induced cell surface PD-L1 protein expression. Cultured tumor cells were transfected with 100 pmol of the indicated siRNAs and were treated 2 days later with IFN-g 250 U/ml. Total and phosphorylated STAT proteins were detected by Western blotting after 15 min of IFN-g treatment, and flow cytometry for cell surface PD-L1 was conducted 1 day later. 397mel expressed HLA-DR constitutively, and this was not affected by STAT knockdown (**c**). **d** and **e.** In JHU-022 cultured SCCHN cells, STAT1 knockdown reduced IFN-g-induced but not constitutive (“no cytokine”) cell surface PD-L1 protein expression. IFN-g also induced HLA-DR expression on JHU-022, which was reduced by STAT1 but not STAT3 knockdown. Percentages represent reduction in total PD-L1 or HLA-DR expression with STAT1 knockdown compared to scrambled siRNA control; numbers in parentheses represent reduction in the amount of PD-L1 or HLA-DR expression that was induced by IFN-g above “no cytokine” baseline expression. Data in panels B-E are representative of 6 tumor lines (4 MELs and 2 SCCHNs). No trans, no transfection; Pos. Ctr., positive control cell lines, mixture of equal amounts of IFN-treated PC-3 cells as pSTAT1 positive control and IL-6-treated COS-7 cells as pSTAT3 positive control; Scrambled, non-specific siRNA mixture

IFN-g is known to signal through the transcription factor STAT1 [24]. However, STAT3 phosphorylation after binding of IFN-g to its receptor has also been reported [25]. To evaluate the potential roles of STAT1 and/or STAT3 activation in mediating PD-L1 protein expression, 31 tumor cell lines (16 MELs, 12 RCCs, 3 SCCHNs) were treated with IFN-g or IL-6, a prototypical STAT3 activator, and then assessed for STAT1 and STAT3 phosphorylation by Western blotting. Results showed that IFN-g induced substantial STAT1 and minor STAT3 phosphorylation in these cultured tumors (*p* < 0.0001 and *p* < 0.0018, respectively). Conversely, IL-6 induced substantial STAT3 and minor STAT1 phosphorylation in the same cell lines (p < 0.0001 and *p* < 0.0101, respectively) (Fig. 2a). However, in contrast to IFN-g, IL-6 induced minimal PD-L1 protein expression on only 2 of 32 tumor lines tested (not shown). To further explore the potential roles of STAT1 and STAT3 in IFN-g-induced PD-L1 expression on tumor cells, we knocked down their expression with specific siRNAs. STAT1, but not STAT3 knockdown reduced IFN-g-induced PD-L1 protein expression by 32–70% in 6 cell lines tested (2 representative examples are shown in Fig. 2b-e). Notably, constitutive PD-L1 expression was not affected by STAT1 or STAT3 knockdown in two SCCHNs and three RCCs tested (a representative example is shown in Fig. 2e, “no cytokine” condition), suggesting that constitutive PD-L1 expression is sustained by mechanisms distinct from cytokine-induced expression. HLA-DR, another IFN-g-inducible molecule, was used as a control in these experiments. Among a total of 6 tumor cell lines assessed, which all showed reduction of IFN-g-induced PD-L1 expression after STAT1 knockdown, three also showed reduction of IFN-g-induced HLA-DR expression, regardless of baseline HLA-DR expression (e.g., JHU-022, Fig. 2e). None showed reduction of constitutive HLA-DR expression (e.g., 397mel, Fig. 2c). This is consistent with the notion that mechanisms regulating IFN-g-induced PD-L1 and HLA-DR expression are only partially overlapping.

### IL-1a and IL-27 induce PD-L1 expression on tumor cells, associated with new PD-L1 mRNA transcription

**Fig. 3:**
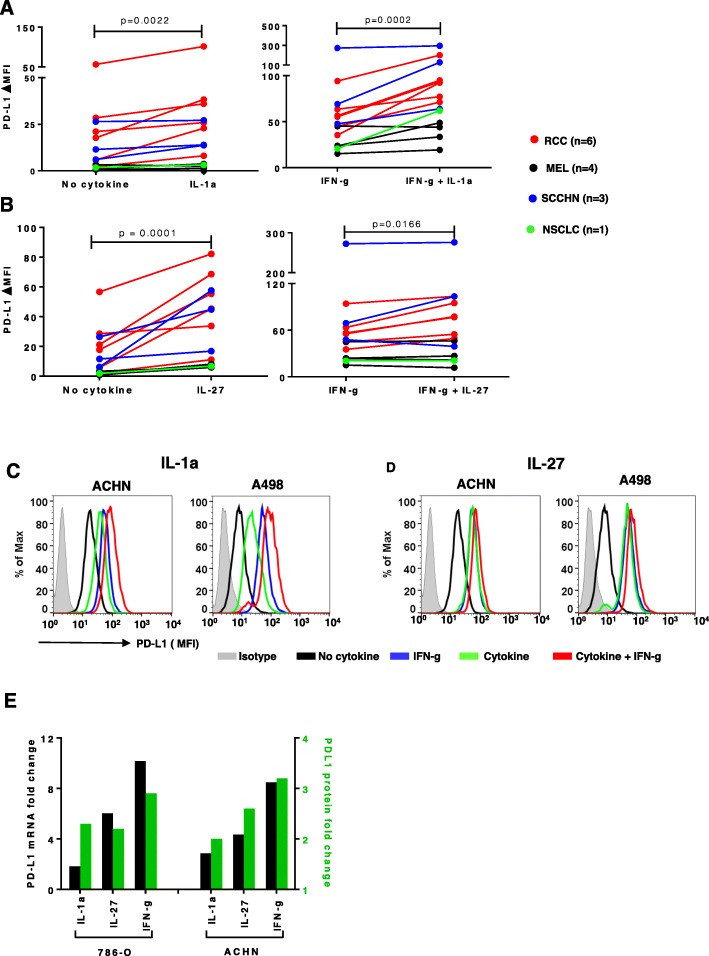
IL-1a- and IL-27-induced PD-L1 protein expression are associated with new PD-L1 mRNA transcription in tumor cells. Fourteen cultured tumor lines were treated with IL-1a (10 ng/ml) or IL-27 (50 ng/ml) for 48 h, and cell surface PD-L1 protein was detected by flow cytometry. **a.** IL-1a alone (left panel) or in combination with IFN-g (right panel) increased PD-L1 expression on tumor cells. ΔMFI, mean fluorescence intensity of PD-L1 staining – isotype control staining. Wilcoxon matched-pairs signed rank test, 2-sided *p*-values**. b.** IL-27 independently increased PD-L1 protein expression on tumor cells (left panel)**,** and a further increase was observed when IL-27 was combined with IFN-g (right panel). **c.** Overlay of flow cytometry histograms from two representative RCC cell lines (ACHN and A498). Either IL-1a or IFN-g independently increased PD-L1 expression, and a greater increase was observed when these cytokines were combined. Note that ACHN and A498 both show constitutive PD-L1 expression in the absence of cytokine treatment. **d.** Overlay of flow cytometry histograms of ACHN and A498 cells treated with IL-27 or IFN-g, alone or in combination. **e.** Increased PD-L1 protein expression induced by IL-1a or IL-27 was associated with new *PDL1* mRNA transcription in 2 RCCs tested. PD-L1 mRNA and cell surface protein were measured by qRT-PCR and flow cytometry at 16 h or 48 h after cytokine exposure, respectively

We previously reported that IL-1a and IL-27 can independently induce PD-L1 protein expression on short-term cultured human Monos [14]. In the current study, we tested the ability of these cytokines to induce PD-L1 on tumor cells. Both IL-1a and IL-27 independently and significantly enhanced or induced PD-L1 protein expression on some cultured tumor cell lines, and further increased IFN-g-induced PD-L1 expression in some cases (Fig. 3a & c, and Fig. 3b & d, respectively; Additional file 8: Table S4). IL-1a increased PD-L1 protein expression by ≥5 MFI in 6 of 14 tumor cell lines tested; notably, the effect of combining IL-1a with IFN-g was more than additive in 12 of 14 tumor cell lines, suggesting the cooperation of distinct signaling pathways (Additional file 8: Table S4). In contrast, while IL-27 alone increased PD-L1 expression by ≥5 MFI in a greater number of cell lines than did IL-1a (11 of 14), the effect of combining IL-27 with IFN-g exceeded IFN-g alone in only 7 cases and was more than additive in only one instance, suggesting that IL-27 and IFN-g signal through a shared pathway (Additional file 8: Table S4). To investigate the selective effects of IL-1a and IL-27 on certain tumor cell lines, we quantified mRNA expression for the subunits of the IL-1a (*IL1R1, IL1RAP*) and IL-27 receptors (*IL27RA, IL6ST*). Expression of these subunits was generally robust among 9 tumor cell lines tested and did not significantly correlate with cytokine-enhanced PD-L1 protein levels (*p* ≥ 0.05, Spearman correlation test; data not shown), suggesting the importance of downstream events in driving PD-L1 expression.

Similar to our findings with IFN-g, changes in PD-L1 protein expression induced by IL-1a or IL-27 corresponded with changes in *PDL1* gene expression, in 2 of 2 RCC lines tested (Fig. 3e). This suggests that new mRNA transcription mediated by IL-1a or IL-27 exposure contributes to PD-L1 regulation. In contrast to the findings described above, the Th17 cytokines IL-17A and IL-23, which we previously detected in the microenvironment of some human cancers but which did not enhance PD-L1 protein expression on Monos [14], also failed to induce PD-L1 on tumor cells (not shown).

### p65 and STAT1 respectively mediate IL-1a- and IL-27-induced PD-L1 expression on tumor cells

**Fig. 4:**
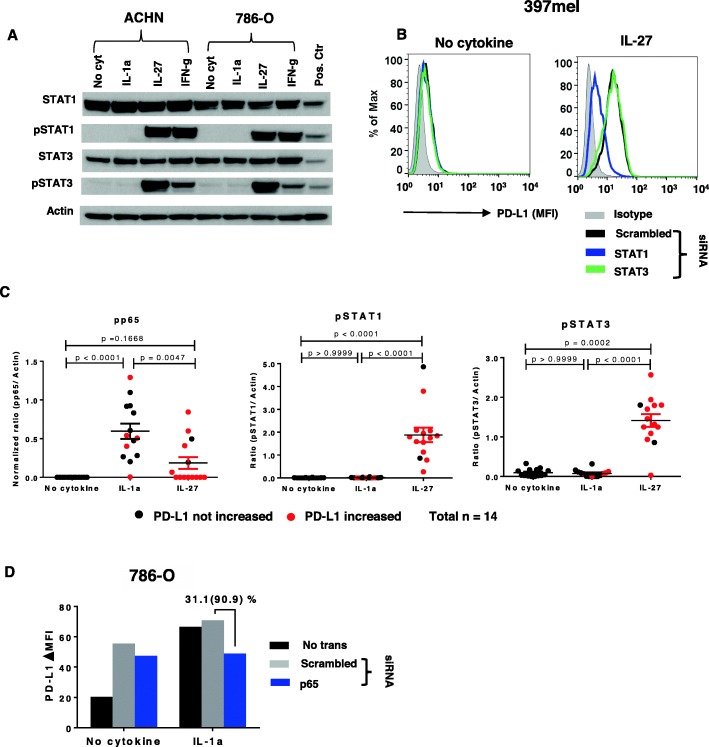
p65 and STAT1 are involved in IL-1a- and IL-27-induced PD-L1 expression, respectively, in tumor cells. Cultured tumor cells were treated with IL-1a (10 ng/ml), IL-27 (50 ng/ml), or IFN-g (100 IU/ml). STAT1, STAT3, and p65 phosphorylation was detected by Western blotting 15 min after cytokine exposure. In experiments to inhibit phosphorylation, transcription factors first were knocked down by transfecting specific siRNAs; after 2 days, transfected cells were treated with cytokines and knockdown effects were assessed with Western blotting. PD-L1 cell surface protein expression was detected by flow cytometry 1 day after cytokine treatment. **a.** In two RCC cell lines, IL-27 exposure caused phosphorylation of both STAT1 and STAT3, while IFN-g selectively phosphorylated STAT1, and IL-1a did not phosphorylate either STAT1 or STAT3. Pos ctr, positive control; mixture of equal amounts of IFN-treated PC-3 cells as a pSTAT1 positive control, and IL-6-treated COS-7 cells as a pSTAT3 positive control. **b**. In 397mel, STAT1 but not STAT3 knockdown significantly reduced IL-27-induced PD-L1 expression. Results representative of 2 tumor cell lines (one MEL, one SCCHN). **c.** IL-1a increased p65 phosphorylation, but not STAT1 or STAT3 phosphorylation, in 14 tumor cell lines. After cytokine exposure, phosphorylation of the indicated transcription factors was detected by Western blotting. Protein bands were quantified by ImageJ and results were normalized to beta-actin expression. Because all cell lines expressed phosphorylated p65 constitutively in the absence of cytokines, values for constitutive normalized ratios have been subtracted from the data depicted for pp65. PD-L1 increased, cytokine-induced enhancement of PD-L1 cell surface expression of ≥5 MFI detected with flow cytometry (red symbols); no or lower levels of PD-L1 enhancement indicated by black symbols. Kruskal-Wallis test (Dunn’s multiple comparisons test), 2-sided p-values. **d.** Knocking down p65 reduced IL-1a-induced PD-L1 protein expression in 786-O. Percentage represents reduction in total PD-L1 expression with p65 knockdown compared to scrambled siRNA control; number in parentheses represents reduction in the amount of PD-L1 expression that was induced by IL-1a above the “no cytokine” baseline expression. Results in panel D are representative of 3 separate experiments with 786-O. Corresponding Western blot is provided in Additional file 2: Fig. S2. ΔMFI, mean fluorescence of specific PD-L1 staining – isotype control staining

To evaluate transcription factors potentially mediating the induction of PD-L1 by IL-1a and IL-27, we assessed phosphorylation of STAT1, STAT3, p65 and c-jun [26, 27]. IL-27 activated STAT1 and STAT3 robustly and equivalently in two RCC cell lines tested, unlike IFN-g which preferentially activated STAT1, and IL-1a which did not activate either transcription factor (Fig. 4a). However, only STAT1 but not STAT3 siRNA knockdown impeded IL-27-induced PD-L1 protein expression (Fig. 4b), consistent with previous reports examining T cells and ovarian cancers [16, 27]. Using the same 14 tumor cell lines that were assessed for the effects of IL-1a and IL-27 on PD-L1 expression as shown in Fig3a and b, respectively, we tested the effects of these cytokines on transcription factor activation. In contrast to IL-27 which significantly activated STAT1 and STAT3 but not p65, IL-1a activated p65 but not STAT1 or STAT3 (Fig. 4c). Interestingly, cell surface PD-L1 expression in the same tumor cells did not correlate with the level of transcriptional activation, suggesting the influence of ancillary signaling events. C-jun, another transcription factor that has been associated in the literature with IL-1a signaling [26], was not significantly activated in these cell lines when compared to no cytokine controls (data not shown). IL-1a-induced PD-L1 protein expression was reduced to baseline levels in the 786-O RCC line by siRNA knockdown of p65 (Fig. 4d; Additional file 2: Figure S2). However, constitutive PD-L1 expression in 786-O was not reduced by p65 knockdown (Fig. 4d, “no cytokine”). In a similar experiment with 397mel, in which IL-1a alone did not induce PD-L1 expression but was synergistic when combined with IFN-g, p65 knockdown reduced PD-L1 levels driven by the cytokine combination by 28% (data not shown). These results suggest that IL-1a signaling drives PD-L1 protein expression through p65, but not STAT1/3, activation.

### PDL1 gene promoter sequence variations do not correlate with quantities of PD-L1 protein induced on tumor cells by IFN-g, IL-1a or IL-27

To determine whether sequence variations in the promoter region of the *PDL1* gene, where transcription factors would be expected to bind, are associated with different levels of tumor cell PD-L1 protein expression induced by cytokines, we sequenced a 650 bp or 2 Kb region upstream of the *PDL1* transcription initiation codon in 33 tumor cell lines and 12 autologous normal tissues. Nine of 33 tumor cell lines harbored -482C and 3 of 33 harbored -382G, which have been reported as SNPs (https://www.ncbi.nlm.nih.gov/snp) [28]. Neither gene alteration correlated with the level of PD-L1 protein expression induced by IFN-g, IL-1a or IL-27 exposure (Additional file 3: Figure S3).

### STAT1 and STAT3 play distinct roles in cytokine-induced PD-L1 expression on monocytes

**Fig. 5:**
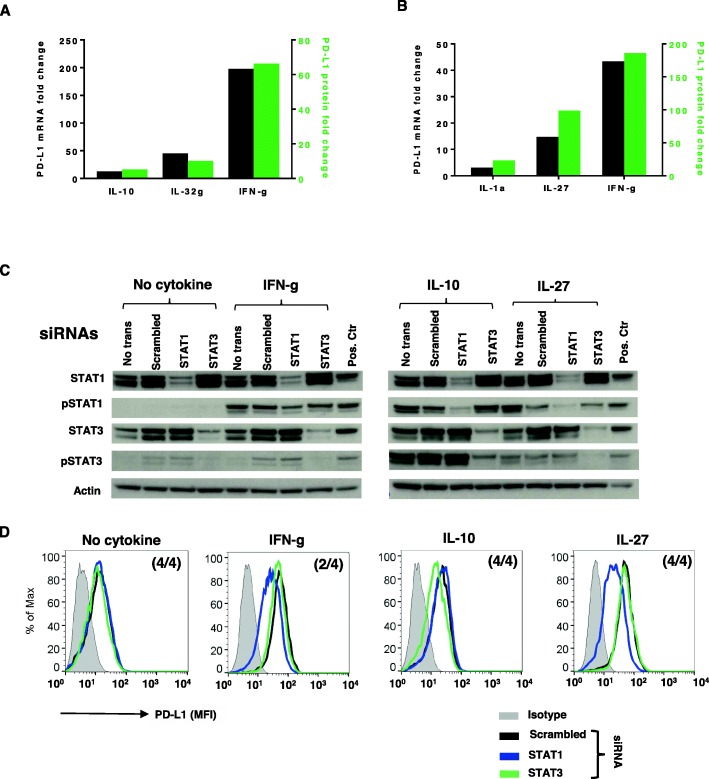
Roles of STAT1 and STAT3 in cytokine-induced PD-L1 protein expression on monocytes. **a** and **b.** Cytokine-induced PD-L1 protein expression on Monos was associated with new *PDL1* mRNA transcription. Monos were treated with IL-1a, IL-10, IL-27, IL-32 g or IFN-g. PD-L1 mRNA and surface protein were measured by q-RT-PCR and flow cytometry after 16 h or 48 h, respectively. Fold changes in PD-L1 protein and mRNA were calculated. Representative data from Monos derived from one of two normal donors are shown. **a.** Fold changes in PD-L1 protein and mRNA levels in normal donor Monos after IL-10 (100 ng/ml), IL-32 g (100 ng/ml) or IFN-g (100 IU/ml) exposure. **b.** Fold changes of PD-L1 protein and mRNA levels in normal donor Monos after IL-1a (10 ng/ml), IL-27 (50 ng/ml) or IFN-g (100 IU/ml) treatment. **c and d.** Fresh isolated Monos were transfected with 300 pmol STAT1 or STAT3 siRNA and treated with the indicated cytokines 2 days later. Total or phosphorylated STATs and cell surface PD-L1 expression were assessed with Western blotting and flow cytometry after 15 min or 1 day, respectively. **c.** siRNA knockdown significantly reduced total and phosphorylated STAT1 and STAT3. **d**. STAT1 knockdown reduced IFN-g- and IL27-induced PD-L1 protein expression, while STAT3 knockdown reduced IL10-induced PD-L1 expression. Numbers in parentheses indicate number of normal donors having Monos with these findings

We have previously reported that IL-1a, IL-10, IL-27 and IL-32 g each increase PD-L1 protein expression on normal human Monos in short-term culture [13, 14]. To test if new mRNA transcription is involved in this response, PD-L1 mRNA and protein were measured in Monos after exposure to each of these four cytokines. For each cytokine tested, changes in *PDL1* mRNA levels accompanied changes in PD-L1 protein expression (Fig. 5a and b). Similar to our findings in tumor cell lines, IFN-g preferentially activated STAT1 in Monos, while IL-27 activated both STAT1 and STAT3; IL-10 preferentially activated STAT3 (Fig. 5c). STAT1 and STAT3 were successfully knocked down in Monos by their respective siRNAs. Knockdown of STAT1, but not STAT3 in Monos from 2 to 4 donors reduced IFN-g- and IL-27-induced PD-L1 protein expression (Fig. 5d). Conversely, knockdown of STAT3 but not STAT1 in Monos from 4 donors reduced IL-10-induced PD-L1 protein expression to constitutive levels, indicating that STAT3 mediates the effect of IL-10 in enhancing PD-L1 expression on Monos (Fig. 5d). Constitutive PD-L1 expression in monocytes was not effected by either STAT1 or STAT3 knockdown (Fig. 5d, left panel). IL-1a induced p65 phosphorylation in Monos (Additional file 4: Figure S4). However, attempted p65 knockdown in Monos was not effective, therefore, we could not assess its impact on IL-1a-induced PD-L1 protein expression. Transcription factors responsible for IL-32 g-induced PD-L1 expression on Monos could not be identified, due to limited information regarding IL-32 g signaling pathways.

## Discussion

There is currently heightened interest in understanding mechanisms that drive expression of the immunosuppressive ligand PD-L1 in the TME, since the PD-1:PD-L1 pathway is now recognized as a dominant immune checkpoint in cancer. While this pathway has been targeted with some success in cancer therapy, current drug development strategies aim to overcome the failure of many tumors to respond to PD-1 pathway blocking drugs, and to address relapses that can occur following initial tumor regression. PD-L1 can be expressed by diverse cell types in the TME, including tumor, immune and endothelial cells. It is assumed that PD-L1 expression by any cell type in the TME can function locally to dampen antitumor immunity. This assumption has been borne out by the development of several predictive biomarkers for the therapeutic effects of anti-PD-1 drugs, that score PD-L1 protein expression on tumor cells, tumor-infiltrating immune cells, or both [29].

IFN-g secreted by tumor-reactive T cells, signaling through the transcription factor STAT1, is the single major cytokine that induces PD-L1 protein expression. This is associated with the phenomenon of adaptive tumor immune resistance [15]. Here we show that the effect of IFN-g in enhancing PD-L1 expression by tumor cells and Monos occurs as a result of new mRNA transcription, rather than translocation of preexisting intracellular protein stores to the cell surface. We also show that this adaptive phenomenon can increase PD-L1 expression in cells already having constitutive expression. This raises the possibility that drugs targeting STAT1 might be deployed against IFN-g-induced PD-L1 expression, to enhance anti-PD-1 therapies. Furthermore, our data indicate that targeting STAT1 might also mitigate PD-L1 expression induced by IL-27. The broad spectrum of biological roles for STAT1 suggests that it could be difficult to target this factor specifically or selectively in tumor cells. However, a recent report from Cerezo et al. suggests that drugs inhibiting eukaryotic initiation factor (eIF)4A can down-modulate STAT1 transcription in a tumor-selective manner, indirectly reducing PD-L1 expression and mediating tumor regression in murine models [30]. Further, these authors demonstrated in vitro that eIF4A chemical inhibition can decrease IFN-g-inducible PD-L1 expression in cell lines from a variety of human tumor types, including melanoma, breast and colon cancer, suggesting the potential for broad applicability of this approach.

In our previous studies of the TMEs of several different cancer types, we found that elevated levels of transcripts for the cytokines IL-1a, IL-10, IL-27 and IL-32 g, in addition to IFN-g, were associated with PD-L1 protein expression. As shown in the current report, each of these cytokines can induce PD-L1 expression on tumor cells and/or Monos in vitro, although to a lesser extent than IFN-g. Furthermore, some cytokines such as IL-1a and IL-27 can have an additive or synergistic effect on PD-L1 expression when combined with IFN-g (Fig. 3, Additional file 8: Table S4). Here we show that IL-27, similar to IFN-g, induces PD-L1 by activating STAT1. However, IL-10 induces PD-L1 by activating STAT3, and IL-1a by activating the p65 transcription factor. This demonstration of the involvement of distinct signaling pathways in driving PD-L1 expression suggests new strategies for targeting diverse transcription factors, or their upstream cytokines or receptors, to mitigate PD-L1 expression in the TME. For instance, STAT3 inhibitors, which are already in clinical testing, have been proposed to synergize with anti-PD-1/PD-L1 through their immunomodulatory effects, based on data from murine models [31]. Furthermore, because the signaling pathway by which IL-1a drives PD-L1 expression is non-overlapping with IFN-g and IL-27, our findings suggest that genetic defects in tumor cell STAT1 signaling, which can be acquired under the selection pressure of anti-PD-1 therapy [23], would not interfere with the ability of IL-1a to sustain tumor cell expression of PD-L1. Such tumors would maintain the ability to evade immune attack from PD-1+ T cells. Ongoing efforts to compare the immune microenvironments of tumors that are responsive or resistant to anti-PD-1 therapies will explore these hypotheses.

Finally, there appears to be a unique set of cytokines, including IL-10 and IL-32 g, which are capable of promoting PD-L1 expression on Monos but not on tumor cells, as studied in our previous report [13] and in unpublished data. The failure of tumor cells to express the IL-10 receptor may explain the failure of IL-10 to promote PD-L1 expression on them (data not shown). Regarding IL-32 g, because its receptor has not yet been identified, potential mechanisms underlying its Mono-selective PD-L1-inducing activity are unknown at this time. PD-L1 expression by Monos may be an important source of immunosuppression in the TME, and antibodies blocking cytokines or cytokine receptors mediating this expression should be considered as potential adjuncts to PD-1 pathway blockade [32].

## Conclusions

Factors driving the expression of the immunosuppressive ligand PD-L1 in the TME are diverse and can vary according to cell type. Both tumor and immune cells are important sources of PD-L1 expression. Cytokines regulating PD-L1 expression, including IFN-g, IL-1a, IL-10, IL-27 and IL-32 g, signal through diverse transcription factors and have variable effects on tumor cells and Monos. Understanding the complex mechanisms underlying intratumoral PD-L1 expression will open new opportunities for developing rationally targeted combination therapies to enhance the effects of anti-PD-1 drugs.

## Supplementary information

**Additional file 1: Figure S1.** New PD-L1 mRNA and protein synthesis are required for IFN-g- induced PD-L1 surface expression on melanoma cells.

**Additional file 2: Figure S2.** IL-1a-induced phosphorylation of p65 is inhibited by p65 knockdown.

**Additional file 3: Figure S3.** Alterations in the *PDL1* promoter region do not correlate with constitutive or cytokine-induced PD-L1 expression on tumor cells.

**Additional file 4: Figure S4.** IL-1a induces phosphorylation of p65 in monocytes from normal donors.

**Additional file 5: Table S1.** Thirty-four tumor cell lines used in this study.

**Additional file 6: Table S2.** Cytokines used in this study.

**Additional file 7: Table S3.** IFN-g-induced PD-L2 cell surface expression on 21 tumor cell lines.

**Additional file 8: Table S4.** Cytokine-induced PD-L1 expression on 14 tumor cell lines.

## Data Availability

All data generated or analyzed during this study are included in this published article and its supplementary information files.

## Abbreviations

ActD: actinomycin D
cHL: classical Hodgkin lymphoma
CHX: cycloheximide
EBV: Epstein-Barr virus
MEL: melanoma
Monos: monocytes
NSCLC: non-small-cell lung carcinoma
PD-L1: programmed death-ligand 1
qRT-PCR: quantitative reverse transcriptase polymerase chain reaction
RCC: renal cell carcinoma
SCCHN: squamous cell carcinoma of head and neck
STAT: Signal transducer and activator of transcription
TME: tumor microenvironment
