# Supplementary material for: Mechanisms regulating PD-L1 expression on tumor and immune cells
Source: J Immunother Cancer. 2019 Nov 15;7:305. doi: 10.1186/s40425-019-0770-2 (PMC6858680; doi:10.1186/s40425-019-0770-2)
Supplement: Supplementary file 1 — Additional file 1: Figure S1. New PD-L1 mRNA and protein synthesis are required for IFN-g- induced PD-L1 surface expression on melanoma cells. [file 40425_2019_770_MOESM1_ESM.pdf]

Figure S1

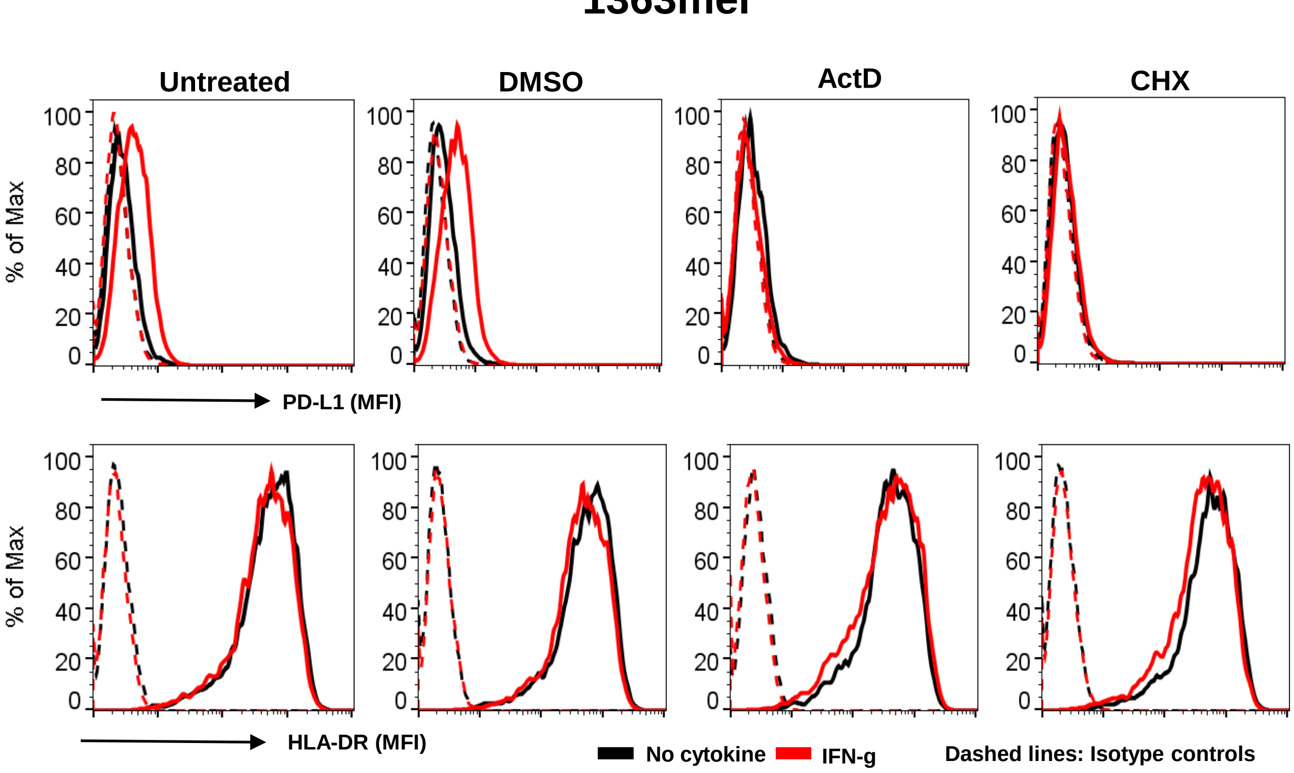

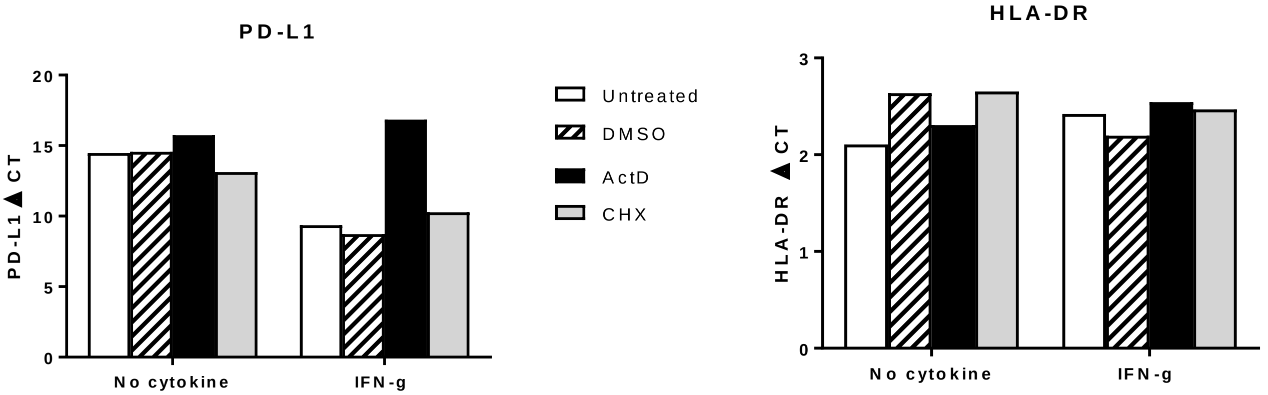

Supplementary Figure S1. New PD-L1 mRNA and protein synthesis are required for IFN-g-induced PD-L1 cell surface expression on melanoma cells. **A.** ActD and CHX completely blocked PD-L1 protein emergence on the surface of melanoma lines after IFN-g exposure. Cultured melanoma cells were preincubated with ActD 10 ug/ml or CHX 2 ug/ml for 1 hr, then incubated with IFN-g 250 U/ml for 6 hr. PD-L1 surface protein was measured by flow cytometry. **B.** ActD, but not CHX, inhibited new IFN-g-induced *PDL1* mRNA transcription. Melanoma lines were treated as described in A. mRNA was measured by qRT-PCR. Ct, cycle threshold. ΔCt = Ct *PDL1* – Ct *GAPDH* housekeeping gene. The lower the ΔCt, the greater *PDL1* mRNA expression. As a control, constitutive HLA-DR expression on the same cells was not affected by either ActD or CHX. Representative data from 1 of 2 melanoma cell lines are shown.
