# Supplementary material for: Mechanisms regulating PD-L1 expression on tumor and immune cells
Source: J Immunother Cancer. 2019 Nov 15;7:305. doi: 10.1186/s40425-019-0770-2 (PMC6858680; doi:10.1186/s40425-019-0770-2)
Supplement: Supplementary file 2 — Additional file 2: Figure S2. IL-1a-induced phosphorylation of p65 is inhibited by p65 knockdown. [file 40425_2019_770_MOESM2_ESM.pdf]

Figure S2

A

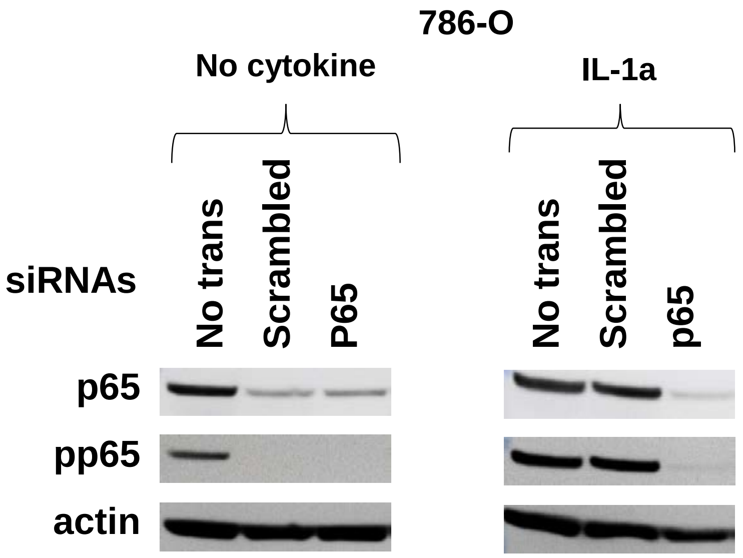

B

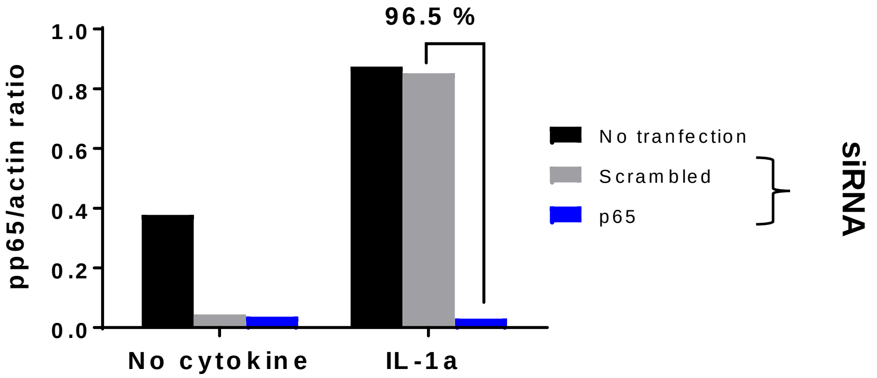

Figure S2. IL-1a-induced phosphorylation of p65 is inhibited by p65 knockdown. Cultured 786-O RCC cells were transfected with siRNA targeting p65, or non-specific scrambled siRNA, then exposed to IL-1a two days later. General and phosphorylated pp65 were detected by Western blotting 15 min after IL-1a exposure. **A.** Western blotting images show a reduction in general and phosphorylated p65 proteins with p65 knockdown. **B.** p65 knockdown reduced phosphorylated p65 by 96.5%, compared to knockdown with the scrambled siRNA control, calculated based on actin-normalized Western blot band intensity (see Methods). These data correspond to the experiment shown in Figure 4D.
