# Supplementary material for: Mechanisms regulating PD-L1 expression on tumor and immune cells
Source: J Immunother Cancer. 2019 Nov 15;7:305. doi: 10.1186/s40425-019-0770-2 (PMC6858680; doi:10.1186/s40425-019-0770-2)
Supplement: Supplementary file 3 — Additional file 3: Figure S3. Alterations in the PDL1 promoter region do not correlate with constitutive or cytokine-induced PD-L1 expression on tumor cells. [file 40425_2019_770_MOESM3_ESM.pdf]

Figure S3

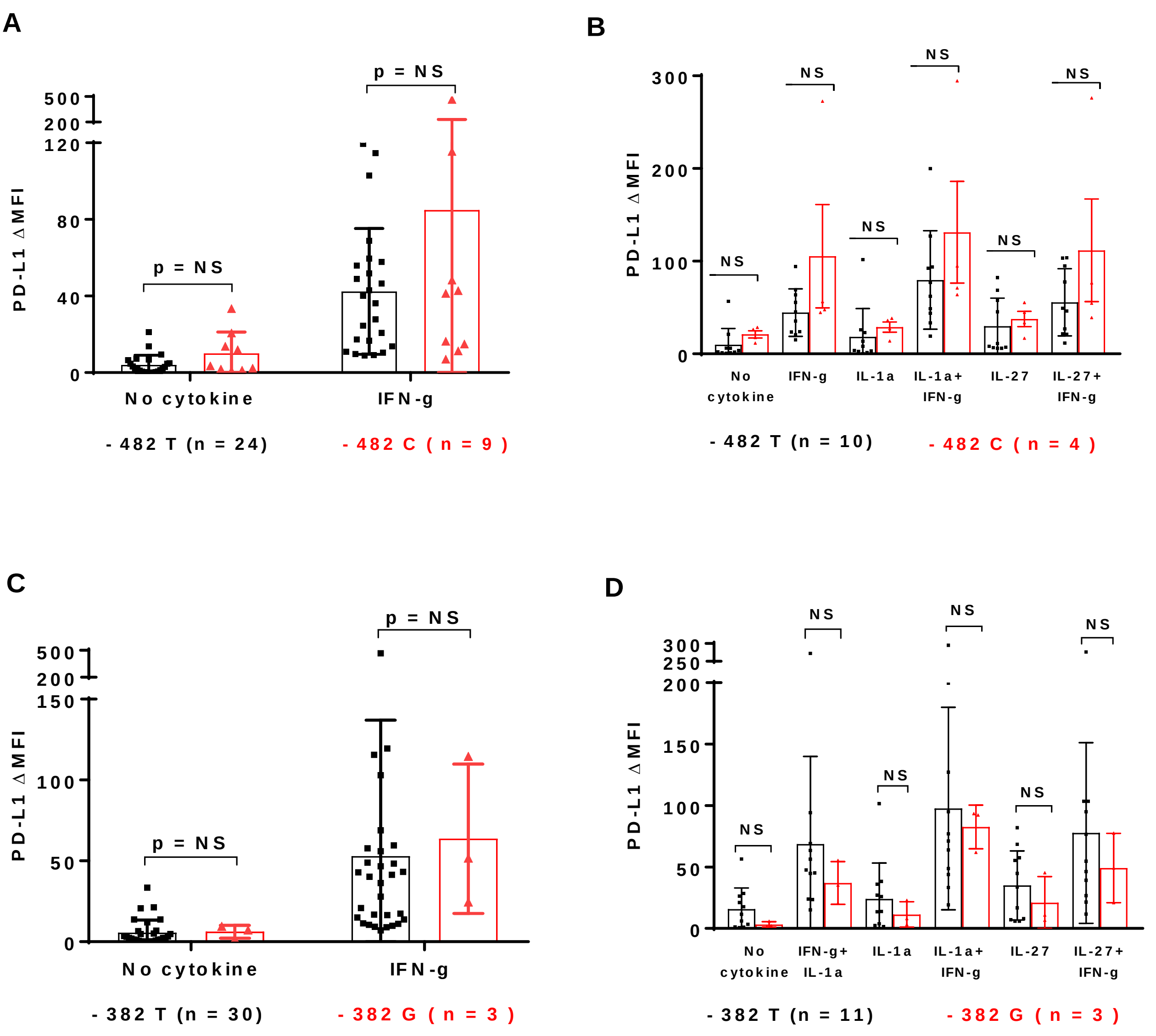

Figure S3. Alterations in the *PDL1* promoter region do not correlate with constitutive or cytokine-induced PD-L1 expression on tumor cells. Cultured tumor cells were exposed to IFN-g (250 U/ml), IL-1a (10 ng/ml,) IL-27 (50 ng/ml), or the indicated cytokine combinations. Cell surface expression of PD-L1 protein was detected by flow cytometry. The proximal promoter region of the *PDL1* gene in each of the 33 tumor lines was sequenced. **A.** The T/C polymorphism at nucleotide -482 does not correlate with IFN-g-induced PD-L1 expression. **B.** The T/C polymorphism at nucleotide -482 does not correlate with IL-1a, IL-27, or combination cytokine-induced PD-L1 expression. **C.** The T/G alteration at nucleotide -382 does not correlate with IFN-g induced PD-L1 expression. **D.** The T/G alteration at nucleotide -382 does not correlate with IL-1a, IL-27, or combination cytokine-induced PD-L1 expression. Mann-Whitney test, 2-sided p-values. NS, not significant, p-value > 0.05.
