# Supplementary material for: Mechanisms regulating PD-L1 expression on tumor and immune cells
Source: J Immunother Cancer. 2019 Nov 15;7:305. doi: 10.1186/s40425-019-0770-2 (PMC6858680; doi:10.1186/s40425-019-0770-2)
Supplement: Supplementary file 4 — Additional file 4: Figure S4. IL-1a induces phosphorylation of p65 in monocytes from normal donors. [file 40425_2019_770_MOESM4_ESM.pdf]

Figure S4

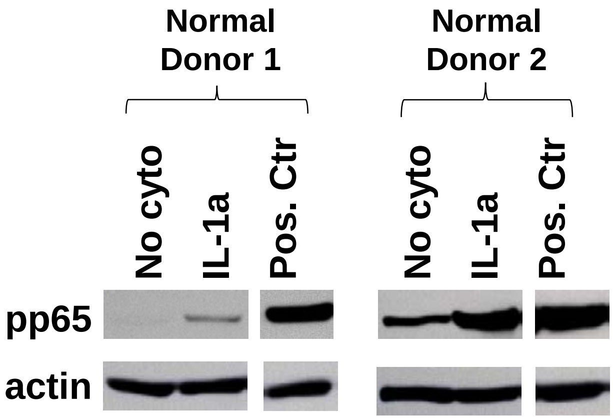

Figure S4. IL-1a induces phosphorylation of p65 in monocytes from normal donors. Freshly enriched Monos from 2 normal donors were treated with IL-1a, and phosphorylation of p65 was detected by Western blotting 15 minutes later. Cyto, cytokine; Pos. Ctr, whole protein lysate from untreated HeLa cells as a positive control for phosphorylated p65 (pp65). Beta-actin was detected as a control for protein loading.
