# Supplementary material for: Mechanisms regulating PD-L1 expression on tumor and immune cells
Source: J Immunother Cancer. 2019 Nov 15;7:305. doi: 10.1186/s40425-019-0770-2 (PMC6858680; doi:10.1186/s40425-019-0770-2)
Supplement: Supplementary file 5 — Additional file 5: Table S1. Thirty-four tumor cell lines used in this study. [file 40425_2019_770_MOESM5_ESM.pdf]

Table S1. Thirty-four tumor cell lines used in this study

Cancer type	Cell line	Number	Source	Reference
**MEL**	397mel, 526mel, 537mel, 553Bmel, 586mel, 624mel, 888mel, 938mel, 1011mel, 1088mel, 1102mel, 1350mel, 1363mel, 1558mel, 1844mel, 1898mel, 2048mel, 2104mel	18	NIH/NCI	[1, 2]
**RCC**	786-O, 1764R*, 2192R*, 2193R*, A498, ACHN, Caki-1, RXF-393, SN12C, TK-10, UO-31, UOK 171*	12	ATCC and * NIH/NCI	[3]
**HNSCC**	JHU-011, JHU-022, JHU-029	3	JHU	[4]
**NSCLC**	A549	1	ATCC	[5]

Supplementary References

1. Topalian SL, Solomon D, Rosenberg SA. Tumor-specific cytolysis by lymphocytes infiltrating human melanomas. J Immunol. 1989;142(10):3714-25.
2. Rodic N, Anders RA, Eshleman JR, Lin MT, Xu H, Kim JH, et al. PD-L1 expression in melanocytic lesions does not correlate with the BRAF V600E mutation. Cancer Immunol Res. 2015;3(2):110-5.
3. Inozume T, Hanada K, Wang QJ, Yang JC. IL-17 secreted by tumor reactive T cells induces IL-8 release by human renal cancer cells. J Immunother. 2009;32(2):109-17.
4. Lyford-Pike S, Peng S, Young GD, Taube JM, Westra WH, Akpeng B, et al. Evidence for a role of the PD-1:PD-L1 pathway in immune resistance of HPV-associated head and neck squamous cell carcinoma. Cancer Res. 2013;73(6):1733-41.
5. Smith BT. Cell line A549: a model system for the study of alveolar type II cell function. Am Rev Respir Dis. 1977;115(2):285-93.
