# Supplementary material for: Mechanisms regulating PD-L1 expression on tumor and immune cells
Source: J Immunother Cancer. 2019 Nov 15;7:305. doi: 10.1186/s40425-019-0770-2 (PMC6858680; doi:10.1186/s40425-019-0770-2)
Supplement: Supplementary file 6 — Additional file 6: Table S2. Cytokines used in this study. [file 40425_2019_770_MOESM6_ESM.pdf]

Table S2. Cytokines used in this study

Cytokine	Major TF(s)	Dose; exposure time*	Main cellular sources	Immune-related functions	Ref
**IFN-g**	STAT1	100 or 250 U/ml; 15 min* or 1, 3, 6,16, 24, 48, 72 hr	T cells and NK cells	Promotes the development and activation of Th1 cells, chemoattraction and activation of monocytes and macrophages, upregulation of antigen presentation molecules and immunoglobulin class switching in B cells, inhibits Th2 and Th17 development	[6, 7]
**IL-1a**	p65, c-jun	10 ng/ml; 15 min* or 24 or 48 hr	macrophages and epithelial cells	T-cell activation, macrophage activation	[8]
**IL-10**	STAT3	100 ng/ml; 15 min* or 24 or 48hr	T cells, monocytes, macrophages, DCs and B cells, and all leukocytes	Promotes phagocytic uptake, suppresses monocytes/macrophages, Th1 and Th17.	[9]
**IL-27**	STAT1	50 ng/ml; 15 min* or 24h or 48hr	monocytes, endothelial cells and dendritic cells	Limits Treg growth and survival, inhibits Th2 and Th17 development, inhibits DC antigen presentation, promotes CD8+ T cell effector functions.	[10, 11]
**IL-32g**	not known	100 ng/ml; 15 min* or 24 or 48hr	monocytes, macrophages	Regulates IFN-g, TNF-a, and IL-6 expression in monocytes, suppresses HIV replication.	[12]

TF: transcription factor.

*Exposure time to assess TF phosphorylation on Western blotting.

Supplementary References

6. Schroder K, Hertzog PJ, Ravasi T, Hume DA. Interferon-gamma: an overview of signals, mechanisms and functions. J Leukoc Biol. 2004;75(2):163-89.
7. Green DS, Young HA, Valencia JC. Current prospects of type II interferon gamma signaling and autoimmunity. J Biol Chem. 2017;292(34):13925-33.
8. Garlanda C, Dinarello CA, Mantovani A. The interleukin-1 family: back to the future. Immunity. 2013;39(6):1003-18.
9. Sabat R, Grutz G, Warszawska K, Kirsch S, Witte E, Wolk K, et al. Biology of interleukin-10. Cytokine Growth Factor Rev. 2010;21(5):331-44.
10. Yoshida H, Hunter CA. The immunobiology of interleukin-27. Annu Rev Immunol. 2015;33:417-43.
11. Villarino AV, Huang E, Hunter CA. Understanding the pro- and anti-inflammatory properties of IL-27. J Immunol. 2004;173(2):715-20.
12. Joosten LA, Heinhuis B, Netea MG, Dinarello CA. Novel insights into the biology of interleukin-32. Cell Mol Life Sci. 2013;70(20):3883-92.
