# Supplementary material for: Mechanisms regulating PD-L1 expression on tumor and immune cells
Source: J Immunother Cancer. 2019 Nov 15;7:305. doi: 10.1186/s40425-019-0770-2 (PMC6858680; doi:10.1186/s40425-019-0770-2)
Supplement: Supplementary file 7 — Additional file 7: Table S3. IFN-g-induced PD-L2 cell surface expression on 21 tumor cell lines. [file 40425_2019_770_MOESM7_ESM.pdf]

Table S3. IFN-g-induced PD-L2 cell surface expression on 21 tumor cell lines

		PD-L2 ΔMFI[a]		PD-L2 ΔΔMFI[d]
		Untreated[b]	IFN-g[c]	
MEL (n = 6)	397mel	0.0	8.4	8.4
	537mel	0.0	5.2	5.2
	938mel	0.0	7.6	7.6
	1102mel	0.0	0.0	0.0
	1844mel	0.4	0.6	0.2
	2048mel	2.4	10.4	8.0
SCCHN (n = 3)	JHU-011	2.1	33.6	31.5
	JHU-022	0.0	19.4	19.4
	JHU-029	0.6	38.9	38.3
RCC (n = 12)	786-O	1.8	12.0	10.2
	1764R	0.5	5.2	4.7
	2192R	0.2	1.3	1.1
	2193R	0.0	3.0	3.0
	A498	0.0	10.4	10.4
	ACHN	1.9	9.5	7.6
	Caki-1	0.0	0.0	0.0
	RXF393	0.0	0.0	0.0
	SN12C	0.2	4.2	4.0
	TK-10	0.0	1.4	1.4
	UO-31	6.9	46.8	39.9
	UOK 171	4.5	23.3	18.8

[a] ΔMFI, mean fluorescence intensity of PD-L2 staning minus isotype control staining.

[b] No cytokine treatment, constitutive PD-L2 expression.

[c] IFN-g, 250 or 500 IU/ml, 48 or 72 hr.

[d] ΔΔMFI, IFN-g-induced PD-L2 ΔMFI minus constitutive PD-L2 ΔMFI.
