# Supplementary material for: Mechanisms regulating PD-L1 expression on tumor and immune cells
Source: J Immunother Cancer. 2019 Nov 15;7:305. doi: 10.1186/s40425-019-0770-2 (PMC6858680; doi:10.1186/s40425-019-0770-2)
Supplement: Supplementary file 8 — Additional file 8: Table S4. Cytokine-induced PD-L1 expression on 14 tumor cell lines. [file 40425_2019_770_MOESM8_ESM.pdf]

Table S4. Cytokine-induced PD-L1 expression on 14 tumor cell lines

			IFN-g		IL-1a		IL-1a +IFN-g		IL-27		IL-27 + IFN-g	
Tumor type	Tumor cell line	No cytokine (ΔMFI[a])	ΔMFI	IFN-g-induced PD-L1 (ΔΔMFI[b])	ΔMFI	IL-1a-induced PD-L1 (ΔΔMFI)	ΔMFI	IL-1a + IFN-g-induced PD-L1 (ΔΔMFI)	ΔMFI	IL-27-induced PD-L1 (ΔΔMFI)	ΔMFI	IL-27 + IFN-g-induced PD-L1 (ΔΔMFI)
MEL (n = 4)	537mel	0.7	23.5	22.8	1.4	0.7	48.7	48.0	7.0	6.3	21.5	20.8
	1011mel	3.3	23.8	20.5	2.5	-0.8	33.4	30.1	5.9	2.6	26.8	23.5
	1102mel	1.2	45.2	44.0	0.0	-1.2	43.9	42.7	8.0	6.8	46.2	45.0
	1363mel	1.1	15.1	14.0	3.7	2.6	19.2	18.1	5.9	4.8	11.6	10.5
HNSCC (n = 3)	JHU-011	26.4	272.5	246.1	27.1	0.7	294.7	268.3	44.8	18.4	276.2	249.8
	JHU-022	11.5	47.6	36.1	13.9	2.4	63.9	52.4	16.8	5.3	39.2	27.7
	JHU-029	6.1	69.0	62.9	13.7	7.6	127.2	121.1	57.5	51.4	103.4	97.3
NSCLC (n = 1)	A549	1.8	20.6	18.8	3.1	1.3	62.0	60.2	6.7	4.9	21.0	19.2
RCC (n = 6)	786-O	56.6	94.2	37.6	101.6	45.0	199.8	143.2	82.1	25.5	103.5	46.9
	A498	5.8	55.3	49.5	22.9	17.1	93.5	87.7	45.3	39.5	77.5	71.7
	ACHN	17.7	56.4	38.7	38.3	20.6	94.9	77.2	55.4	37.7	76.7	59.0
	TK-10	2.2	35.3	33.1	8.1	5.9	92.3	90.1	11.0	8.8	49.2	47.0
	UO-31	21.1	63.5	42.4	25.9	4.8	77.1	56.0	68.5	47.4	94.9	73.8
	UOK 171	28.5	44.7	16.2	36.0	7.5	71.2	42.7	33.7	5.2	54.7	26.2

[a] ΔMFI, mean fluorescence intensity of PD-L1 staning minus isotype control staining.

[b] ΔΔMFI, single cytokine-induced-PD-L1 ΔMFI minus no cytokine PD-L1 ΔMFI, or combination cytokine-induced PD-L1 ΔMFI minus no cytokine PD-L1 ΔMFI.

Yellow shading, PD-L1 induced by the cytokine combination is more than 5 MFI greater than adding the effects of the individual cytokines.
